# Pathway size matters: the influence of pathway granularity on over-representation (enrichment analysis) statistics

**DOI:** 10.1186/s12864-021-07502-8

**Published:** 2021-03-16

**Authors:** Peter D. Karp, Peter E. Midford, Ron Caspi, Arkady Khodursky

**Affiliations:** 1 Bioinformatics Research Group, SRI International, 333 Ravenswood Drive, Menlo Park, 94025 CA USA; 2 Department of Biochemistry, Molecular Biology, and Biophysics, University of Minnesota, 140 Gortner Lab, 1479 Gortner Ave, Saint Paul, 55198 MN USA

**Keywords:** Metabolomics, Enrichment analysis, Over-representation analysis, Pathways, Pathway size, BioCyc, EcoCyc, KEGG

## Abstract

**Background:**

Enrichment or over-representation analysis is a common method used in bioinformatics studies of transcriptomics, metabolomics, and microbiome datasets. The key idea behind enrichment analysis is: given a set of significantly expressed genes (or metabolites), use that set to infer a smaller set of perturbed biological pathways or processes, in which those genes (or metabolites) play a role. Enrichment computations rely on collections of defined biological pathways and/or processes, which are usually drawn from pathway databases. Although practitioners of enrichment analysis take great care to employ statistical corrections (e.g., for multiple testing), they appear unaware that enrichment results are quite sensitive to the pathway definitions that the calculation uses.

**Results:**

We show that alternative pathway definitions can alter enrichment *p*-values by up to nine orders of magnitude, whereas statistical corrections typically alter enrichment *p*-values by only two orders of magnitude. We present multiple examples where the smaller pathway definitions used in the EcoCyc database produces stronger enrichment *p*-values than the much larger pathway definitions used in the KEGG database; we demonstrate that to attain a given enrichment *p*-value, KEGG-based enrichment analyses require 1.3–2.0 times as many significantly expressed genes as does EcoCyc-based enrichment analyses. The large pathways in KEGG are problematic for another reason: they blur together multiple (as many as 21) biological processes. When such a KEGG pathway receives a high enrichment *p*-value, which of its component processes is perturbed is unclear, and thus the biological conclusions drawn from enrichment of large pathways are also in question.

**Conclusions:**

The choice of pathway database used in enrichment analyses can have a much stronger effect on the enrichment results than the statistical corrections used in these analyses.

**Supplementary Information:**

The online version contains supplementary material available at (10.1186/s12864-021-07502-8).

## Background

Pathway analysis has become a popular way to analyze gene expression data. This family of analysis methods seeks to find which biological processes have changed their activity levels most significantly across two different biological states [1]. Pathway analysis is also used for interpreting metabolomics data, where again researchers use pathways to map alterations in the levels of individual metabolites to changes in biological processes. Pathway analysis has also become popular in analysis of microbiome datasets: researchers calculate the abundances of different pathways across different microbiome samples to seek correlations between the presence of different biological processes with phenotypic differences such as diseased versus normal populations.

Researchers have explored a number of mathematical methods for calculating pathway activity levels and pathway abundances, but all of these methods ultimately depend on a collection of pathways within pathway databases (DBs) such as BioCyc [2], KEGG [3], and Reactome [4] (some methods also use the biological processes defined in Gene Ontology [5]). BioCyc is a collection of 18,000 pathway databases including the EcoCyc [6] database for *Escherichia coli*. Although this article uses example pathways from EcoCyc, the BioCyc databases for other organisms contain pathways of similar size and hence we would expect similar results to apply when comparing them to KEGG.

It has been noted previously that pathway DBs differ significantly in their content, both in how they conceptualize pathways and in the genes and reactions present in specific pathways [7, 8]. The first question we ask in this article is: to what degree does the choice of pathway database affect the results returned by a pathway-analysis method?

The second question we investigate is: If a given pathway has a high enrichment score (or abundance score, for the microbiome), what does this result tell us biologically? That is, what have we learned about the biological system under study? For example, does the pathway clearly correspond to one biological process, or does the pathway integrate so many biological processes that the biological significance of identifying that pathway as enriched is of little meaning? We show that the answers to Questions 1 and 2 depend on the pathway database being used.

For Question 1, we investigate the differences between EcoCyc and KEGG using one of the oldest and most popular pathway-analysis methods, enrichment analysis of gene-expression data using the hypergeometric distribution (also called over-representation analysis) [1]. The main input to the enrichment-analysis method is a set of those genes from a gene-expression experiment that are significantly differentially regulated over some threshold across two experimental conditions of interest. The output of enrichment analysis is a set of enriched pathways, and an enrichment score (*p*-value) for each. The degree to which a pathway *P* is considered to be enriched depends on two factors: (1) how many genes in *P* are present in the input list of significantly differentially regulated genes, and (2) how many total genes does *P* contain? After all, if 3 genes from *P* were present in the significant-genes set, we would consider that much more significant if *P* contained 4 total genes than if *P* contained 15 total genes. The enrichment *p*-value for a pathway indicates the probability that the set of significantly expressed genes of a given size would have occurred by chance. In over-representation analysis, unlike in the gene set enrichment analysis, the set by itself is irrelevant, only its size matters.

Several approaches are used to calculate enrichment scores [9], but all of them compare two ratios in one way or another. For the case of pathway enrichment, the first ratio is the number of significantly expressed genes in a particular pathway to the total number of genes in the pathway. The second ratio is the total number of significantly expressed genes to the total number of genes assigned to any pathway.

For a given set of observed genes, assuming equivalent pathways in each database cover the same subset of the observed genes, the enrichment score will still depend on the number of genes in the pathway (the denominator of the first ratio) as well as the total number of genes that are assigned to pathways in the database (the denominator of the second ratio).

Although methods based on binomial distributions or chi-squared tests have been used, tests based on the hypergeometric distribution are the most popular. As the hypergeometric is a discrete distribution, a one-tailed statistic is the sum of probability mass functions calculated at a set of values equal to or more extreme than the value of interest. The probability mass function is:

$$P(k) = \frac{\binom{K}{k}\binom{N-K}{n-k}}{\binom{N}{n}} $$

Where *K* is the total number of significantly expressed genes, *k* is the number of significantly expressed genes in the pathway of interest, *n* is the total number of genes in the pathway, and *N* is the total number of (pathway associated) genes in the database.

For enrichment, the statistic of interest is the probability of observing *k* or more significantly expressed genes in the pathway by chance. This is the sum of the mass functions for each gene count that is greater than or equal to the number of genes observed:

$$P(x \ge k) = \sum_{k=0}^{x}\frac{\binom{K}{k}\binom{N-K}{n-k}}{\binom{N}{n}} $$

To address Question 1 we perform an enrichment analysis on corresponding EcoCyc and KEGG pathways using the same set of input genes and study how different the results are. We consider series of examples where we compute enrichment scores for the same sets of genes across corresponding EcoCyc and KEGG pathways. A second way we address the question is through a mathematical analysis in which we demonstrate the effect of pathway size on *p*-values when other factors in the calculation are fixed. We show that the *p*-value computed by one over-representation analysis method can vary by up to nine orders of magnitude depending on the size of the corresponding EcoCyc and KEGG pathways — EcoCyc and KEGG pathways differ significantly in their average size. This finding is much larger than variations due to multiple-comparison corrections, which are viewed as essential refinements to the statistical methods.

To address Question 2 we analyze several examples where one KEGG pathway contains multiple EcoCyc pathways.

## Results

### Question 1: analytic approach

As detailed in the Appendix in the Additional file 1, for a fixed number of significantly expressed genes, if two pathways are enriched for the same number of genes, the smaller pathway will have a smaller enrichment *p*-value (and thus more significant). Specifically, by expanding the expression for probability mass function, we found that the probability mass function will always have a smaller value for the smaller pathway. Because the final one-tailed *p*-value is a sum of probability mass values for a range of counts of genes, the relative advantage of the smaller pathway increases as the number of significantly expressed genes increases.

### Question 1: comparing enrichment scores across example pathways

We selected six pairings of EcoCyc and KEGG pathways, listed in Table 1. Four of these are pairings of amino acid synthesis pathways and two are purine and pyrimidine metabolism pathways and indicates the number of genes associated with each pathway.

**Table 1:** Pairs of pathways selected as examples

EcoCyc Pathway	KEGG Pathway	Pathway Size		
		EcoCyc	KEGG	Table
L-cysteine biosynthesis CYSTSYN-PWY	Cysteine and Methionine metabolism map00270	3	34	2
Arginine biosynthesis ARGSYN-PWY	Arginine biosynthesis map00220	12	18	3
L-selenocysteine biosynthesis I PWY0-901	Selenocompound metabolism map00450	4	17	4
L-valine biosynthesis VALSYN-PWY	Valine, leucine, and isoleucine biosynthesis map00290	9	16	5
Guanosine deoxyribosenucleotides de novo biosynthesis II PWY-7222	Purine Metabolism map00230	10	78	6
Pyrimidine deoxyribonucleotides de novo biosynthesis PWY-7184	Pyrimidine Metabolism map00240	13	51	7

The first four pairs are amino acid synthesis pathways. The remaining pairs are two large KEGG pathways covering purine and pyrimidine metabolism and one of several EcoCyc pathways that correspond to a part of the pathway. Each row contains corresponding EcoCyc and KEGG pathways identified by their name and EcoCyc or KEGG pathway identifier, and the pathway size expressed as number of associated genes

Tables 2, 3, 4 and 5 compare enrichment calculations for corresponding pairs of EcoCyc and KEGG pathways. In each table we vary the number of genes present for the enrichment calculation (that is, the number of genes whose expression changed significantly in a hypothetical gene-expression experiment), and compute an enrichment score for the EcoCyc pathway and for the KEGG pathway. For example, in Table 2 we compare an EcoCyc pathway containing 3 genes (L-cysteine biosynthesis) to a KEGG pathway containing 34 genes (Cysteine and Methionine Biosynthesis). That KEGG pathway is the closest biological equivalent to the EcoCyc pathway, and contains the EcoCyc pathway as a component. This size difference between EcoCyc and KEGG pathways is quite common — in each case we compared the closest biological pathways. Each table considers from 1 to *N* genes where *N* is the number of genes in the smaller pathway (which is always the EcoCyc pathway).

**Table 2:** Comparing *p*-values for EcoCyc CYSTSYN-PWY and KEGG map00270

Count of Significantly Expressed Genes	EcoCyc *p*-value	Corrected for Multiple Comparisons	Native KEGG *p*-value	Corrected for Multiple Comparisons
3	4.6×10^−9^	1.6×10^−6^	7.5×10^−6^	8.9×10^−4^
2	5.0×10^−6^	1.8×10^−3^	3.9×10^−4^	4.7×10^−2^
1	2.7×10^−3^	9.7×10^−1^	2.0×10^−2^	1.0

Each row starts with the number of significantly expressed genes in a data set. Corrected *p*-values to the right of each *p*-value column use Bonferroni as a “worst case” correction as discussed in the text

**Table 3:** Comparing *p*-values for EcoCyc ARGSYN-PWY and KEGG map00220

Count of Significantly Expressed Genes	EcoCyc *p*-value	Corrected for Multiple Comparisons	Native KEGG *p*-value	Corrected for Multiple Comparisons
12	1.7×10^−28^	6.0×10^−26^	1.8×10^−26^	2.1×10^−24^
11	1.8×10^−25^	6.5×10^−23^	4.2×10^−24^	5.0×10^−22^
10	1.0×10^−22^	3.5×10^−20^	8.8×10^−22^	1.0×10^−19^
9	3.6×10^−20^	1.3×10^−17^	1.6×10^−19^	1.9×10^−17^
8	9.8×10^−18^	3.5×10^−15^	2.7×10^−17^	3.3×10^−15^
7	2.1×10^−15^	7.6×10^−13^	4.2×10^−15^	5.0×10^−13^
6	3.9×10^−13^	1.4×10^−10^	5.9×10^−13^	7.0×10^−11^
5	6.1×10^−11^	2.1×10^−8^	7.6×10^−11^	9.0×10^−9^
4	8.3×10^−9^	2.9×10^−6^	9.1×10^−9^	1.1×10^−6^
3	1.0×10^−6^	3.6×10^−4^	1.0×10^−6^	1.2×10^−4^
2	1.1×10^−4^	3.9×10^−2^	1.1×10^−4^	1.3×10^−2^
1	1.1×10^−2^	1.0	1.1×10^−2^	1.0

Corrected *p*-values to the right of each *p*-value column use Bonferroni as a “worst case” correction as discussed in the text

**Table 4:** Comparing *p*-values for EcoCyc PWY0-901 and KEGG map00450

Count of Significantly Expressed Genes	EcoCyc *p*-value	Corrected for Multiple Comparisons	Native KEGG *p*-value	Corrected for Multiple Comparisons
4	1.7×10^−11^	5.9×10^−9^	7.2×10^−9^	8.5×10^−7^
3	1.8×10^−8^	6.5×10^−6^	8.5×10^−7^	1.0×10^−4^
2	1.0×10^−5^	3.5×10^−3^	9.6×10^−5^	1.1×10^−2^
1	3.6×10^−3^	1.0	1.0×10^−2^	1.0

Corrected *p*-values to the right of each *p*-value column use Bonferroni as a “worst case” correction as discussed in the text

**Table 5:** Comparing *p*-values for EcoCyc VALSYN-PWY and KEGG map00290

Count of Significantly Expressed Genes	EcoCyc *p*-value	Corrected for Multiple Comparisons	Native KEGG *p*-value	Corrected for Multiple Comparisons
9	1.6×10^−22^	5.8×10^−20^	3.9×10^−20^	4.6×10^−18^
8	1.8×10^−19^	6.3×10^−17^	8.1×10^−18^	9.6×10^−16^
7	9.7×10^−17^	3.4×10^−14^	1.5×10^−15^	1.8×10^−13^
6	3.5×10^−14^	1.3×10^−11^	2.5×10^−13^	3.0×10^−11^
5	9.6×10^−12^	3.4×10^−9^	3.9×10^−11^	4.6×10^−9^
4	2.1×10^−9^	7.5×10^−7^	5.4×10^−9^	6.5×10^−7^
3	3.8×10^−7^	1.4×10^−4^	7.0×10^−7^	8.4×10^−5^
2	6.0×10^−5^	2.1×10^−2^	8.4×10^−5^	1.0×10^−2^
1	8.2×10^−3^	1.0	9.4×10^−3^	1.0

Corrected *p*-values use Bonferroni as a ’worst case’ correction as discussed in the text

Tables 6 and 7 are similar, but illustrate more extreme differences in pathway size. KEGG has large pathways that correspond to purine (map00230) and pyramidine (map00240) metabolism. These pathways overlap several EcoCyc pathways of a range of sizes. The results displayed are for the largest EcoCyc pathways overlapped, which ironically shows the largest difference in *p*-values.

**Table 6:** Comparing *p*-values for EcoCyc Guanosine deoxyribonucleotides de novo biosynthesis II (PWY-7222) and KEGG map00230

Count of Significantly Expressed Genes	EcoCyc *p*-value	Corrected for Multiple Comparisons	Native KEGG *p*-value	Corrected for Multiple Comparisons
10	1.51×10^−24^	5.35×10^−22^	2.53×10^−14^	3.01×10^−12^
9	1.64×10^−21^	5.82×10^−19^	6.14×10^−13^	7.31×10^−11^
8	8.94×10^−19^	3.16×10^−16^	1.47×10^−11^	1.75×10^−9^
7	3.25×10^−16^	1.15×10^−13^	3.48×10^−10^	4.14×10^−8^
6	8.84×10^−14^	3.13×10^−11^	8.12×10^−9^	9.67×10^−7^
5	1.93×10^−11^	6.83×10^−9^	1.87×10^−7^	2.23×10^−5^
4	3.51×10^−9^	1.24×10^−6^	4.25×10^−6^	5.06×10^−4^
3	5.48×10^−7^	1.94×10^−4^	9.54×10^−5^	1.14×10^−2^
2	7.50×10^−5^	2.65×10^−2^	2.11×10^−3^	2.52×10^−1^
1	9.12×10^−3^	1.0	4.63×10^−2^	1.00

Corrected *p*-values use Bonferroni as a ’worst case’ correction as discussed in the text

**Table 7:** Comparing *p*-values for EcoCyc pyrimidine deoxyribonucleotides de novo biosynthesis I (PWY-7184) and KEGG map00240

Count of Significantly Expressed Genes	EcoCyc *p*-value	Corrected for Multiple Comparisons	Native KEGG *p*-value	Corrected for Multiple Comparisons
13	2.03×10^−30^	7.19×10^−28^	3.49×10^−21^	4.16×10^−19^
12	2.20×10^−27^	7.79×10^−25^	1.50×10^−19^	1.78×10^−17^
11	1.19×10^−24^	4.23×10^−22^	6.28×10^−18^	7.47×10^−16^
10	4.32×10^−22^	1.53×10^−19^	2.57×10^−16^	3.05×10^−14^
9	1.18×10^−19^	4.16×10^−17^	1.02×10^−14^	1.22×10^−12^
8	2.56×10^−17^	9.05×10^−15^	4.00×10^−13^	4.76×10^−11^
7	4.64×10^−15^	1.64×10^−12^	1.53×10^−11^	1.82×10^−9^
6	7.23×10^−13^	2.56×10^−10^	5.70×10^−10^	6.78×10^−8^
5	9.86×10^−11^	3.49×10^−8^	2.08×10^−8^	2.48×10^−6^
4	1.20×10^−8^	4.23×10^−6^	7.45×10^−7^	8.86×10^−5^
3	1.31×10^−6^	4.63×10^−4^	2.61×10^−5^	3.11×10^−3^
2	1.30×10^−4^	4.60×10^−2^	8.98×10^−4^	1.07×10^−1^
1	1.19×10^−2^	1.00	3.02×10^−2^	1.00

Corrected *p*-values use Bonferroni as a ’worst case’ correction as discussed in the text

Recall that the enrichment score depends in part on the total number of genes assigned to pathways within the database. These total numbers differ significantly for EcoCyc and KEGG: EcoCyc assigns 1096 genes to pathways, whereas KEGG reports 1686 *E. coli* genes assigned to pathways (as of August 14, 2019). In Tables 2, 3, 4 and 5 we provide two different *p*-values for the KEGG pathway: column 3 is computed using the actual KEGG total of 1686 genes, whereas to provide an apples-to-apples comparison, column 4 is computed for KEGG using the EcoCyc total of 1096 genes. Why does KEGG assign so many more genes to pathways than does EcoCyc? Most of the differences are due to the fact that one very large KEGG pathway (map02010) is not a pathway at all, it is an enumeration of 134 ABC transporters involving 179 *E. coli* genes. Further, KEGG assigns a number of enzymes to pathways that EcoCyc does not assign to any pathway, usually because EcoCyc considers these enzymes to define connections among pathways rather than defining their own pathway.

In the analysis in this section, we chose to adjust for multiple comparisons using Bonferroni corrections rather than the more common Benjamini-Hochberg method [10]. This approach allowed us to avoid the question of which gene was removed from the starting set at each successive line in the table. The Benjamini-Hochberg method will always penalize the result with the lowest *p*-value by a factor equal to the number of pathways considered, which is equivalent to the adjustment made for all *p*-values by the Bonferroni test. In some cases, a particular subset of genes will actually favor a different pathway with a lower *p*-value; in those instances, using the Bonferroni correction is conservative, but analytically more tractable than considering all possible subsets of genes in these examples.

We used a count of 119 for the number of pathways of *E. coli* in KEGG, and 354 as the number of pathways in EcoCyc. Note that this number is smaller than the number (1080) used in the Bonferroni and Benjamini-Hochberg corrections in the EcoCyc SmartTables and Dashboard. This later value includes all pathway types in the EcoCyc class hierarchy. Since no class hierarchy exists among the KEGG pathways (apart from a small shallow hierarchy in BRITE), we adjusted using the simple count of pathways.

Additional file 5 provides more evidence that KEGG pathways tend to be much larger than BioCyc pathways. For six additional KEGG pathways, we list the MetaCyc pathways that these KEGG pathways contain. MetaCyc [11] contains many non-*E. coli* pathways and is the multi-organism reference pathway database from which pathways are computationally projected when predicting pathways in other BioCyc databases. These six KEGG pathways contain the following number of MetaCyc pathways: 31, 17, 12, 12, 25, 26. We note that MetaCyc pathways whose names differ in the roman numerals they contain are usually pathway variants, meaning the pathways accomplish a similar biological function, and often share some reactions, but they will contain some different reactions.

### Question 2: biological inference from enriched pathways

Imagine that a pathway-enrichment calculation or a pathway-abundance calculation has identified a EcoCyc pathway or a KEGG pathway as highly enriched or as highly abundant in a given biological situation. What does that result tell us biologically?

For a EcoCyc pathway, the result is straightforward: we have learned that the expression of the biological process corresponding to that pathway is perturbed in the situation under study. For example, if the EcoCyc pathway for L-cysteine biosynthesis is highly enriched, then the cellular process of L-cysteine biosynthesis is highly perturbed. If the arginine biosynthesis pathway is highly enriched, then arginine biosynthesis is highly perturbed. The interpretation is so obvious because the vast majority of EcoCyc pathways correspond to a single biological process, one that is often regulated as a unit, and often evolved as a unit. Of course, due to post-transcriptional effects, changes in pathway expression may not yield corresponding changes in pathway activity.

**Fig. 1:**
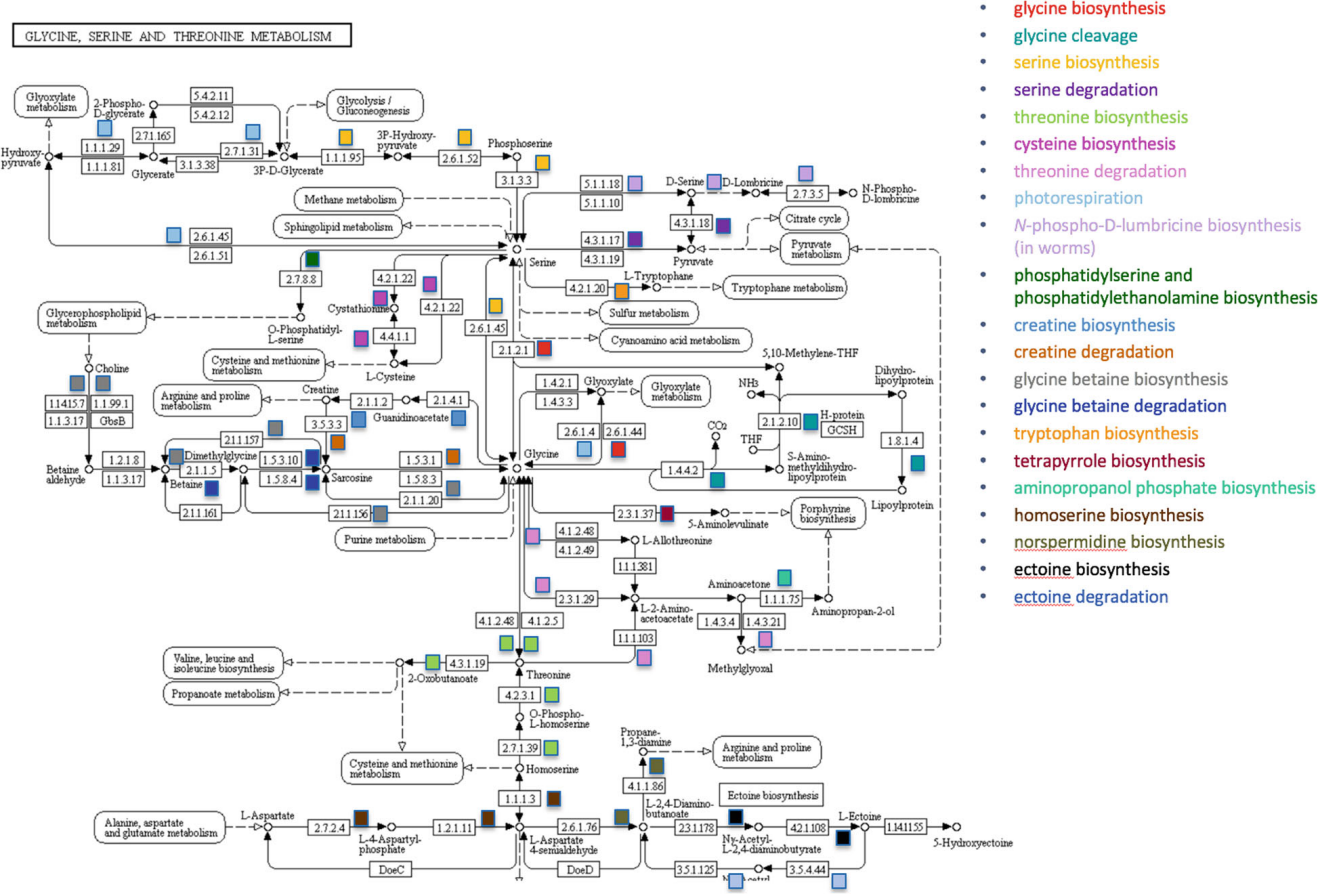
KEGG map00260. The biological processes present in this map are listed along the right. Each process name is color coded, and those colors identify that process within the map. Each colored square is positioned next to a reaction within that process. KEGG diagram from [21], downloaded November 2019

The result is much less straightforward to infer for KEGG pathways, in particular, for KEGG maps. For example, imagine that KEGG map00260, “glycine, serine, and threonine metabolism,” is highly enriched. Have we learned that glycine metabolism is highly perturbed in this biological situation, or that serine metabolism is highly perturbed, or that threonine metabolism is highly perturbed? Or should we conclude that some combination of these three processes are highly perturbed? But it is more complicated than this: the term “serine metabolism” could mean either serine degradation or serine biosynthesis (and in fact both processes are present in map00260) — so now there are six biological processes that might be perturbed: either the biosynthesis or degradation of glycine, serine, and threonine. Figure 1 shows that the situation is even more complicated. KEGG map00260 actually includes the 21 different biological processes listed in the right side of this figure. These processes were manually identified within the KEGG map by reference to the MetaCyc database [11]. Any combination of those 21 different processes could be perturbed to yield an elevated enrichment or abundance score for the pathway. Worse yet, the map could receive a high enrichment or abundance score if enough single genes from each of those individual processes were highly perturbed, even if none of the individual processes were themselves highly perturbed.

A similar pattern is present for the six KEGG pathways in Additional file 5, which contain the following number of MetaCyc pathways: 31, 17, 12, 12, 25, 26.

Thus, because of the large mosaic nature of KEGG maps, it is not at all clear what to conclude if a KEGG map shows a high enrichment score. Any one, or any combination, of multiple biological processes might be perturbed. The mosaic quality of KEGG maps can be useful for some applications, such as for understanding the connectivity between multiple metabolic pathways. But for enrichment analysis, their mosaic-ism is a strong liability.

But KEGG contains a different type of pathway called a *module.* Modules correspond much more closely to EcoCyc pathways and therefore to individual biological processes. Thus, KEGG modules do not suffer from the confusion just illustrated for KEGG maps (although many publications do in fact use KEGG maps for enrichment analysis.) But KEGG modules suffer from a different limitation: they are quite incomplete. KEGG contains only 348 metabolic modules (December 12, 2019) compared to the 2801 pathways in MetaCyc version 24.0 (December 12, 2019). Thus, many biological processes are not covered by KEGG modules, so perturbations to those processes cannot be detected if KEGG modules are used for enrichment analysis.

### Comparing effect of gene set size at fixed **p**-value

Here we determined how differences in the number of significantly expressed genes affects the sensitivity of high-throughput analysis and representation of annotated pathways in high-throughput samples, which is an inverse problem to inferring over-representation on the basis of a *p*-value threshold.

The lower the *p*-values calculated from X genes drawn from a pathway of size Y, the more unlikely it is that at least X genes from a set of size Y could be drawn in a sample of size N by chance. However, the *p*-value, which is a random variable associated with the null distribution, is not a quantitative indicator of the enrichment itself, which is an alternative to the random draw hypothesis. To obtain a quantitative measure of enrichment which could be used in direct comparison between different yet significant outcomes, we fixed a *p*-value at a significant threshold and determined the minimum number of genes from a pathway that would have to be found in a biological sample in order for that pathway to be considered enriched. We called this quantity a critical subset size or a minimally required number of successes. We compared the effects of pathways sizes on the sizes of critical subsets. To that end we enumerated critical subsets for every pathway in EcoCyc and KEGG for sample sizes between 50–500 genes and a typical result is shown for a 100-gene sample (Figure S1 in Additional file 1). The distribution of critical subset sizes for EcoCyc pathways is shifted toward smaller values compared to KEGG. Our results indicate that on average a collection of genes of interest would need to contain two times fewer EcoCyc genes than KEGG genes in order for it to be biologically interpretable. Another characteristic of the EcoCyc distribution is high relative frequency of critical subsets of the same size. This implies that the higher granularity of EcoCyc pathways results in an advantageous statistical property of the annotation — relatively high homogeneity of set sizes (Figure S1 in Additional file 1). That in turn should reduce the effect of set size variation on the rate of false negatives in over-representation analysis, thereby enabling more robust inference.

Although on average EcoCyc pathways and the corresponding critical subsets are smaller than KEGG pathways [7], the truly functionally analogous pathways may possibly be much closer in size (despite the preceding section’s specific examples already providing anecdotal evidence against it) and thus would not have a large effect on the enrichment analysis. To avoid any bias, we defined analogous pathways as unique, highly significantly overlapping, sets of KEGG and EcoCyc genes (Additional file 4), i.e., if several EcoCyc pathway sets could be matched at a comparable *p*-value and identical overlap with one KEGG pathway or vice-versa, we chose the pair of sets with the smallest combined number of genes. We also imposed an additional constraint of at least three genes per EcoCyc pathway. We identified 69 pairs of analogous pathways that satisfied those criteria (Additional file 4). A pairwise comparison of analogous pathways revealed that KEGG sets are on average about 3.5 times larger than the corresponding EcoCyc sets (Figure S2 in Additional file 1). To examine the effect of pathway size differences among the 69 pairs on the size of critical subsets, we calculated the respective ratios of critical subset sizes for every pair of the pathways for sample sizes between 50–500. For an absolute majority of analogous pathways (46 out of 69, 66%), the median size of the critical subset is higher for KEGG than for EcoCyc annotation (Figure S3 in Additional file 1). To illustrate how the critical subset ratio may vary as a function of sample size, we plotted the ratio for a pair of analogous pathways, “EcoCyc: superpathway of S-adenosyl-L-methionine biosynthesis” and “KEGG: eco00270: Cysteine and methionine metabolism,” with a typical median ratio of 2. With the exception of only three sample sizes, for which minimum significant gene-set sizes were the same for both annotations, EcoCyc annotations required fewer genes to meet the *p*-value cutoff (Figure S4 in Additional file 1). Further, we observed that statistically significant over-representation is consistently achieved by a greater proportion of genes in the pathway when using EcoCyc pathways (Figure S5 in Additional file 1). Both advantages could be attributed to a more refined annotation of individual pathways in the EcoCyc database.

## Discussion

Omics assays capture snapshots of the state of the respective transcriptome, proteome, or metabolome. A typical snapshot can be viewed as a point in a geometric space whose dimensionality is defined by the number of measurable components of an ome, such as transcripts, proteins, or metabolites. This dimensionality can be very high, especially relative to the number of snapshots recorded in a given biological experiment. Since most omics assays are performed to gain insight into system physiology, the high dimensionality of the omics observations often complicates interpretation of the findings. However, focusing only on the over-represented subsets in the outcomes of omics experiments can substantially reduce dimensionatlity. As a result, the components making up the subset can be used directly to interpret the omics experiment. This approach, which is known as over-representation, or enrichment analysis, was originally used to interpret transcriptome data [12] and has become one of the standard tools for interpreting high-throughput omics observations [1]. Subsets are derived from pathway and process annotations. Here we present the first examination of how the properties of these annotations may affect the identification of over-represented subsets, and hence, biological interpretation of the results.

The KEGG and EcoCyc annotations of metabolic pathways differ substantially in their granularity [7]. This difference results in size differences between analogous pathways/gene-sets, which in turn affects the outcome of enrichment analyses and ultimately the interpretability of an omics model. Since annotated EcoCyc pathways are smaller than their KEGG counterparts across the board, EcoCyc (and BioCyc) pathway definitions are more likely to result in identification of enriched pathways — meaning pathways that exceed a *p*-value threshold — than are KEGG pathways. We showed this analytically in the Appendix in Additional file 1.

We provide multiple examples of this situation Tables 2, 3, 4, 5, 6 and 7 as can be seen by comparing the multiple-comparison-corrected *p*-values for EcoCyc and KEGG (columns 3 and 5 respectively). The largest differences occur in Tables 6 and 7. For example, the fourth row in Table 7 shows the *p*-values that would result from the presence of ten significantly expressed genes in EcoCyc pathway PWY-7184 versus KEGG pathway map00240: the *p*-values differ by a factor of over five orders of magnitude, even after correction for multiple comparisons: 1.5×10^−19^ for EcoCyc vs 3.1×10^−14^ for KEGG. The first row of the same table shows an uncorrected difference (columns 2 and 4) for 13 significantly expressed genes of nine orders of magnitude (EcoCyc 2.0×10^−30^, versus KEGG 3.5×10^−21^). Such large differences could easily influence the biological findings by results in a pathway being called as significantly enriched or not. The higher a fraction of the genes in a pathway are significantly expressed, the more this difference in *p*-values is amplified.

Put another way, we showed that for KEGG and EcoCyc pathways to attain the same enrichment *p*-value, the KEGG pathway would need to have from 1.3–2.0 times as many significantly expressed genes observed as would the EcoCyc pathway (Figures S1, S3, and S4 in Additional file 1).

Further, the statistically significant enrichment is achieved with fewer genes but at a higher representation rate, which makes the resulting reduction in dimensionality more biologically meaningful and conclusive. For example, two subsets, one represented by 10% of all genes in the set and another by more than 50%, may both be statistically significantly enriched, but the latter pathway is represented much more fully, making the omics observation more interpretable.

For many of the rows in Tables 2, 3, 4 and 5, both the KEGG and the EcoCyc pathways have a *p*-value less than.05. One might therefore argue that our findings have no practical consequences, because given a *p*-value cutoff of.05, there will be no difference in the selected pathways. We disagree with that sentiment for several reasons. First, some investigators may use a cutoff different from.05. Second, for other pathways not shown here, there may be cases where EcoCyc is below the cutoff and KEGG is not. Third, users of enrichment analysis do not simply consider whether a pathway passes the cutoff, they also consider the magnitude of the *p*-value as an indication of the significance of that pathway to the biological effect being studied. When *p*-values differ by nine orders of magnitude, the effect on the user’s perception of the pathway will be profound. Put another way, researchers perform statistical corrections to obtain more accurate *p*-values, and their analyses would be considered delinquent if such corrections were omitted.

As just discussed, the main purpose of enrichment analysis is dimensionality reduction: to winnow the large set of genes or metabolites observed in an omics experiment down to a much smaller set of biological pathways or processes that are perturbed in that experiment. Yet, as shown in Figure 1, very large pathway definitions are a direct impediment to that goal. KEGG map00260 contains 21 different biological processes, ranging from serine degradation to photorespiration to ectoine biosynthesis. If an enrichment analysis resulted in a favorable *p*-value for this pathway, we would not know whether to infer that one of the 21 processes was perturbed, or if some combination of the 21 processes were perturbed, or whether the enrichment score is not meaningful because it resulted from individual genes from many of those pathways when in fact none of the pathways was significantly perturbed on its own. Note that the misleading name of the pathway (“glycine, serine, and threonine metabolism” may lead investigators to incorrectly conclude that 3 of the 21 processes are perturbed.

In related work, Mubeen et al. [13] compared the enrichment results from a set of pathway databases across three different enrichment methods including over-representation. For over-representation, they compared three primary databases (KEGG, Reactome [4], and WikiPathways [14]) and a merged pathway database (MPath) that they generated from the three primary databases. They reported differences in the enrichment (over-representation) analyses for four cancer datasets from The Cancer Genome Atlas (TCGA) [15]. Differences in significance levels were found in each paired comparison of databases, but the differences were not in a consistent direction favoring one database over another. Our results show a consistent difference between EcoCyc and KEGG.

Marco-Ramell et al. [16] compared a collection of tools for enrichment analysis and a collection of databases. The focus in their comparison of databases was number of metabolites included as well as whether the databases were being updated. They did not discuss the effect of size of the pathways in the databases.

## Conclusions

We conclude that pathway size can have a significant impact on the results of enrichment-analysis calculations. We provided several types of evidence to support this conclusion: (1) We showed that direct comparisons of enrichment calculations for sets of significantly expressed genes where only the pathway database was allowed to vary demonstrated that the choice of EcoCyc (or BioCyc for other organisms) versus KEGG could affect the enrichment *p*-value by as many as nine orders of magnitude. (2) We showed that, when holding the enrichment *p*-value constant, 1.3–2.0 times as many significantly expressed genes would be needed to attain that *p*-value for KEGG as for EcoCyc. (3) We showed analytically how *p*-value depends on pathway size. Thus, smaller, more precise pathway definitions can lead to more sensitive enrichment analyses.

We further conclude that pathway size can have a large impact on the interpretability of enrichment calculations. When a large pathway is composed of many individual biological processes, and that pathway receives a strong *p*-value, inferring which biological process is perturbed in the experimental condition of interest, or if *any* biological process is significantly perturbed, is very difficult — the strong *p*-value may result from the perturbation of genes from multiple biological processes where no single process is strongly perturbed.

## Methods

In a usual pathway enrichment analysis, several considerations must be made, such as how to adjust for multiple comparisons, and whether to consider just enrichment (a one-tailed statistical test) or both enrichment and depletion (a two-tailed test). Although the hypergeometric test provides an “exact” *p*-value, it is commonly considered statistically conservative and adjustments such as a mid-range *p*-value [9] are used instead. Because this study’s object is comparing results across databases, the simplest, most analytically tractable test (one-tailed without adjustments for being conservative or multiple comparison) will be presented.

### Comparing enrichment scores across example pathways

We selected four *E. coli* pathways related to amino acid synthesis to serve as examples. The pathways, their corresponding EcoCyc and KEGG entries, and the sizes of each are listed in Table 1. The data is taken from EcoCyc version 23.0 and from KEGG (release 91.0) data downloaded on August 14, 2019. Gene counts for EcoCyc were calculated using the genes-of-pathway lisp function. Gene counts for KEGG were obtained from each KEGG pathway’s web info page. All calculations in this section were performed using EcoCyc’s implementation of the Fisher Exact test (using the lisp function fisher-exact-pvalue).

### Biological inference from enriched pathways

We also conducted an analysis using a fixed *p*-value cut-off and determined the minimum number of significantly expressed genes required to reach that level of significance. The *p*-value significance cutoff for over-representation was calculated from the cumulative hypergeometric distribution and adjusted for the multiplicity of pathways in each of the two annotations (EcoCyc and KEGG). The Benjamini-Hochberg method was used in this analysis to adjust for multiple comparisons. [10]. The critical subset size was determined by lookup using R 4.0.3 and tydiverse (Additional file 2). The KEGG annotation for the comparative analysis of critical subset sizes was obtained from the “gage” Bioconductor package [17].

To obtain a quantitative measure of enrichment which could be used in direct comparison between different yet significant outcomes, we fixed a *p*-value at a significant threshold and determined the minimum number of genes from a pathway that would have to be found in a biological sample in order for that pathway to be considered enriched. We called this quantity a critical subset size. We compared the effects of pathways sizes on critical subset sizes. To that end we enumerated critical subsets for every pathway in EcoCyc and KEGG.

## Supplementary Information

**Additional file 1** Supplementary material as a pdf file.

**Additional file 2** R code to reproduce supplemental figures. Please set your R working directory (e.g., setwd()) to the location of the following two files. You may also need to load package matrixStat (e.g., library(matrixStat)) if this is not in your R environment. This code has been tested in R 4.0.3. It will generate each of the supplemental figures in order.

**Additional file 3** Counts of EocCyc pathway genes used to generate supplement figures.

**Additional file 4** Set of 69 similar KEGG and EcoCyc pathways, used to generate Supplement Figure S2.

**Additional file 5** A listing of six KEGG maps and the multiple MetaCyc pathways those maps correspond to.

## Data Availability

The data used in the comparison of example pathways analysis was collected from EcoCyc version 23.5 at [18] and from KEGG version 91 in August 2019 using the KEGG API (Application Programming Interface) at [19]. Access to the EcoCyc database is available without subscription, though we strongly encourage creating a free user account. We did not use any administrative access to this database. We had no administrative access to the KEGG database, so all information we used was publicly available. Data in the supplementary file kegg-metacyc-pathways.pdf were derived from MetaCyc [20] version 24.5 in KEGG version 96.0 in December 2020. MetaCyc is publicly available on the same terms as EcoCyc. No administrative access was used. Other data are in the following files which are included as supplementary materials.

## Abbreviations

KEGG: Kyoto Encyclopedia of Genes and Genomes
DBs: Databases
TCGA: The Cancer Genome Atlas
API: Application Programming Interface
