# Supplementary material for: Pathway size matters: the influence of pathway granularity on over-representation (enrichment analysis) statistics
Source: BMC Genomics. 2021 Mar 16;22:191. doi: 10.1186/s12864-021-07502-8 (PMC7967953; doi:10.1186/s12864-021-07502-8)
Supplement: Supplementary file 1 — Additional file 1 Supplementary material as a pdf file. [file 12864_2021_7502_MOESM1_ESM.pdf]

Appendix

This appendix proves the claim that if the same set of significantly expressed genes is associated with two pathways of different size, the enrichment p-value will be smaller for the smaller pathway. The hypergeometric distribution is a discrete distribution defined for integer values of n, k and K. The associated p-values are sums of the probability mass function (PMF) for a range of values of k. The PMF for a particular value of k is

$$P(k) = \frac{\binom{K}{k}\binom{N-K}{n-k}}{\binom{N}{n}}$$

with the associated one-tailed p-value (for a random variable x) equal to

$$P(x \geq k) = \sum_{k=0} \frac{\binom{K}{k}\binom{N-K}{n-k}}{\binom{N}{n}}$$

The PMF expression can be rewritten by expanding the binomial coefficient expressions:

$$\frac{\left(\frac{K!}{(K-k)!k!}\right)\left(\frac{(N-K)!}{(N-K-(n-k))!(n-k)!)}\right)}{\left(\frac{N!}{n!(N-n)!}\right)}$$

leading to

$$\frac{K!(N-k)!n!(N-n)!}{k!(K-k)!(n-k)!(N-K-(n-k))!N!}$$

Now consider two over representation tests, with K, N, and k held fixed, and $n_1 < n_2$ being the corresponding n values. Then the ratio of the PMFs for n_1 and n_2 is

$$\frac{\left(\frac{K!(N-k)!n_1!(N-n_1)!}{k!(K-k)!(n_1-k)!(N-K-(n_1-k))!N!}\right)}{\left(\frac{K!(N-k)!n_2!(N-n_2)!}{k!(K-k)!(n_2-k)!(N-K-(n_2-k))!N!}\right)}$$

which simplifies to

$$\frac{(n_2-k)!n_1!}{(n_1-k)!n_2!}$$

Expanding the factorials:

$$\frac{(n_2-k)(n_2-k-1)\cdots(2)(1)}{(n_1-k)(n_1-k-1)\cdots(2)(1)}\frac{n_1(n_1-1)\cdots(2)(1)}{n_2(n_2-1)\cdots(2)(1)}$$

Because $n_2 > n_1$, canceling and simplifying leads to

$$\frac{(n_2 - k) \cdots (n_1 - k + 1)}{n_2 \cdots (n_1 + 1)}$$

or

$$\left(\frac{n_2 - k}{n_2}\right)\left(\frac{n_2 - k - 1}{n_2 - 1}\right)\cdots\left(\frac{n_1 - k + 1}{n_1 + 1}\right)$$

Each term in this expression is less than 1 (for $k > 0$); thus the product is less than 1 and thus the ratio of the original PMF expressions is less than one. The same argument applies for all PMF values that are summed into the p-value for $P(X > k)$; thus the p-value for n_1 is smaller than for n_2.

Supplemental Figures

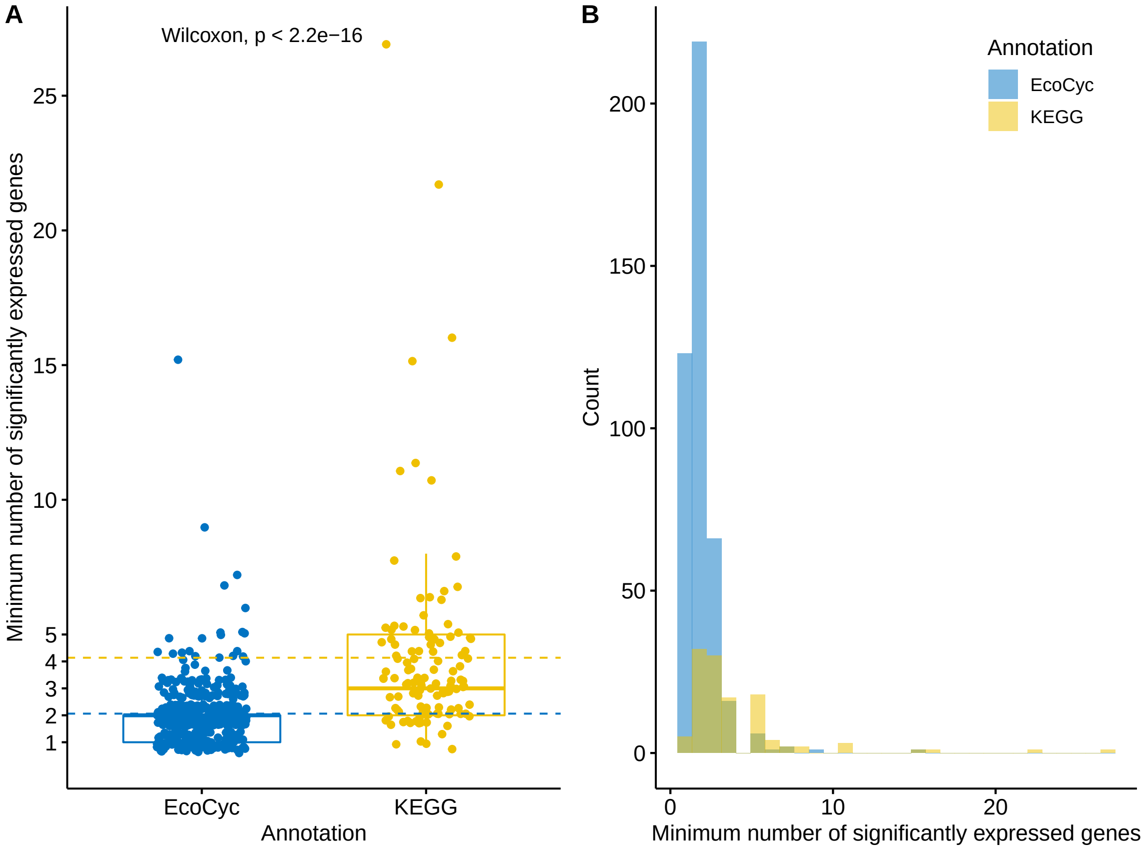

Figure S1: Distributions of critical subset sizes or minimum required numbers of genes for a significant over-representation of KEGG or EcoCyc gene sets. The minimum required number corresponds to the smallest number of successes that satisfies a chance probability (adjusted for multiplicity of measurements by Benjamini-Hochberg correction) cut-off of 0.01 calculated from a hypergeometric distribution with a sample size of 100. A) Boxplots of critical subset sizes: the mean minimum number of significantly expressed gene is significantly lower for EcoCyc annotation than for KEGG. Horizontal dashed lines correspond to the means of distributions. B) An orthogonal view to the one in A); distribution of minimum required number of successes for all 117 KEGG and 435 EcoCyc gene sets.

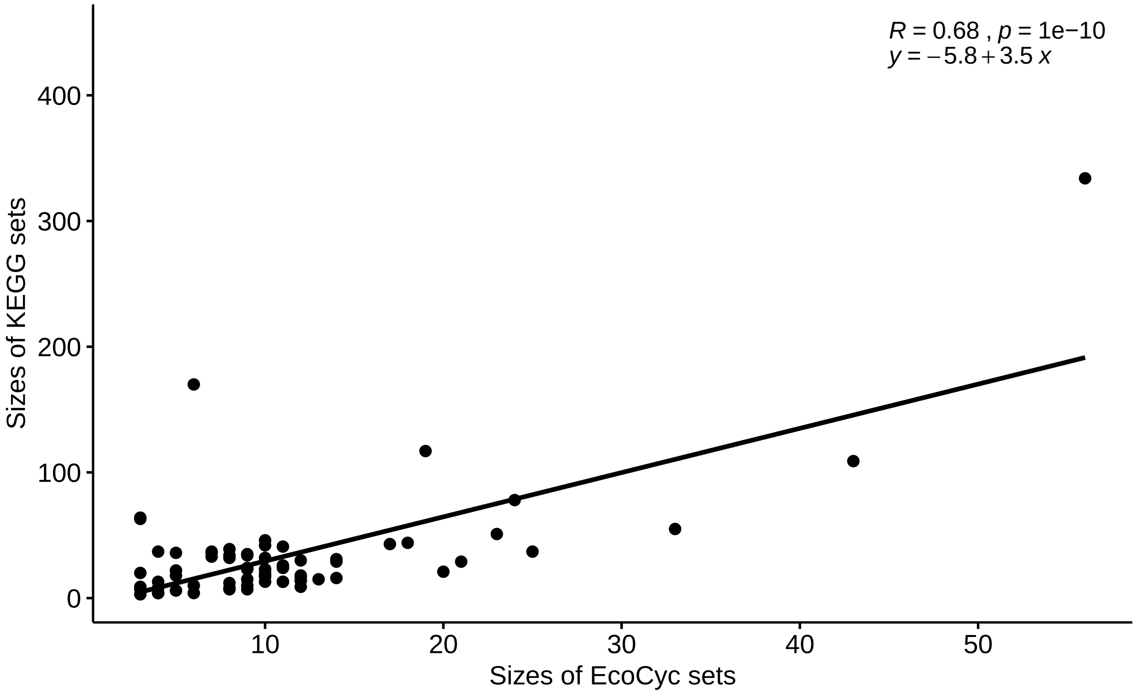

Figure S2: Relationship between sizes of analogous KEGG and EcoCyc pathways. Analogous pathways were identified as described in the text. Each of the 69 analogous pathways has a pair of size values associated with it: one value is from KEGG database, another from EcoCyc. The regression analysis determined the relationship between these values across all 69 pairs.

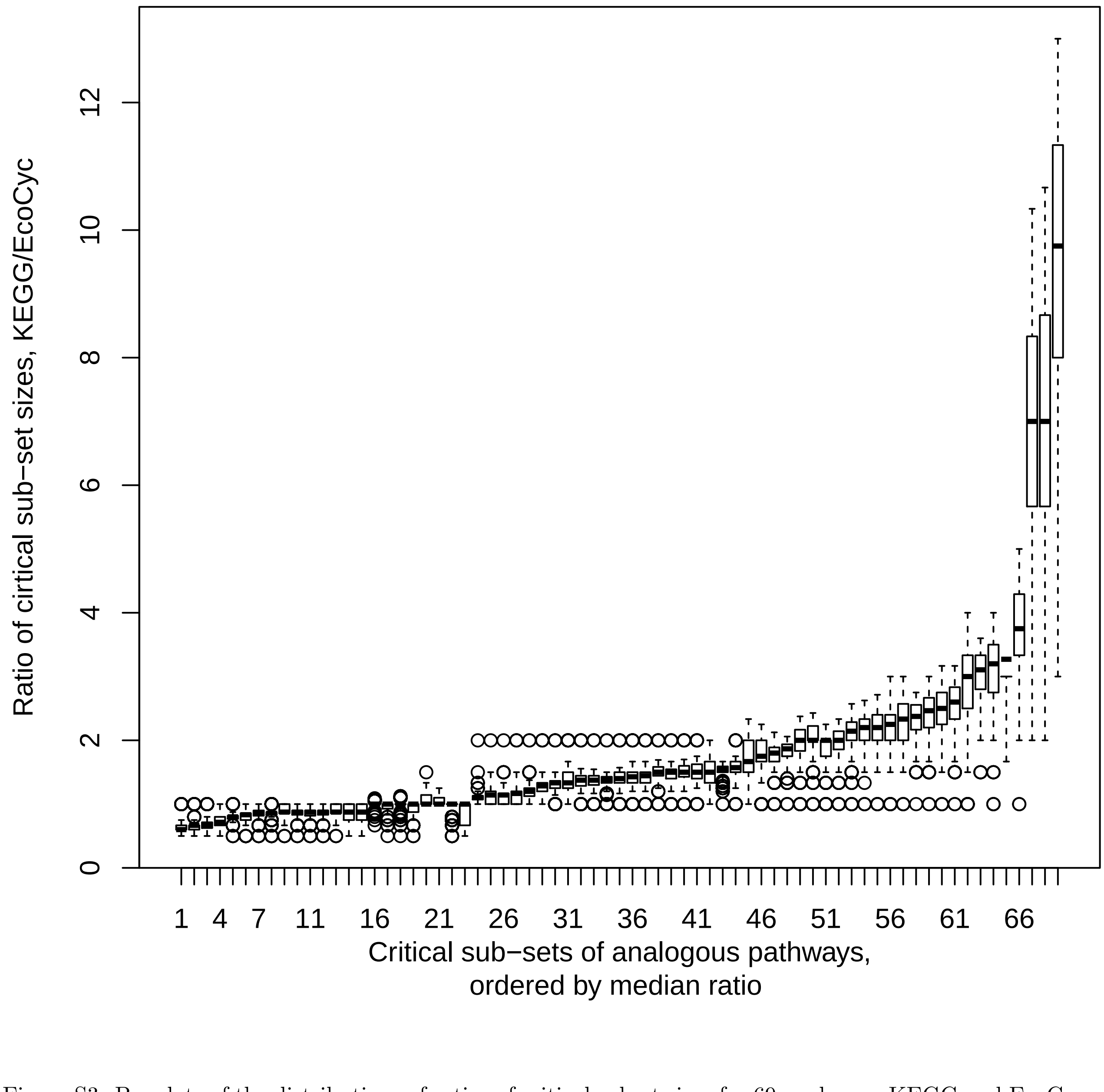

Figure S3: Boxplots of the distributions of ratios of critical subset sizes for 69 analogous KEGG and EcoCyc pathways. Bold horizontal lines correspond to medias, circles depict outlier values.

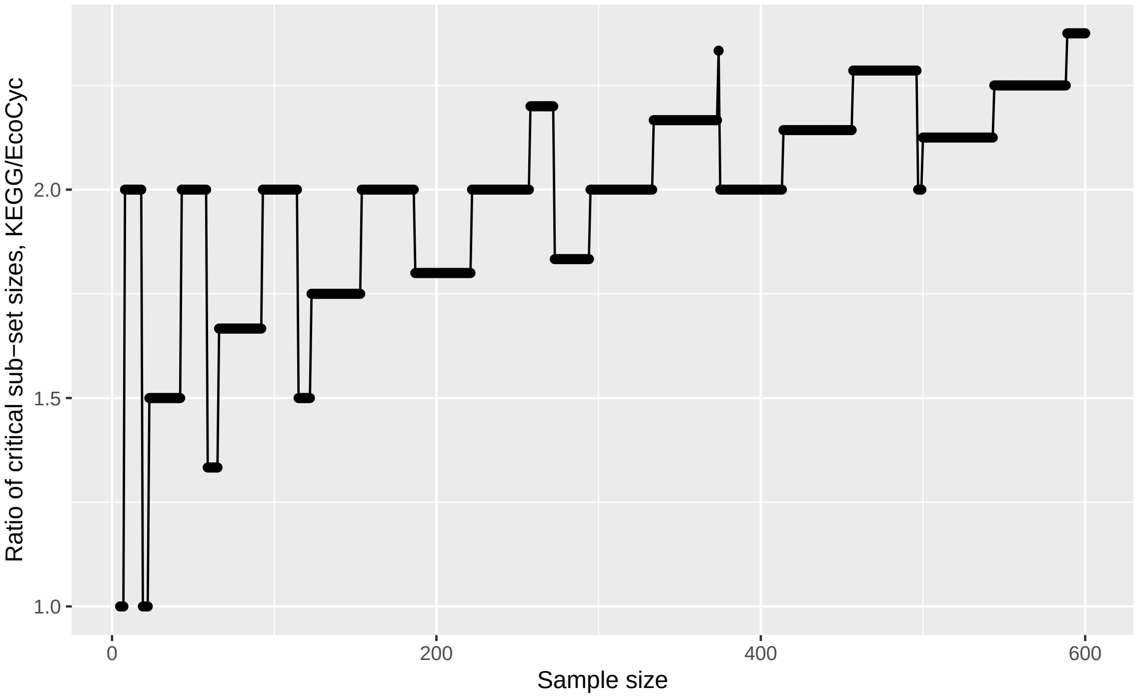

Figure S4: Ratio of critical subset sizes between two analogous KEGG and EcoCyc pathways as a function of sample size. Critical subsets for "EcoCyc: superpathway of S-adenosyl-L-methionine biosynthesis" and "KEGG: eco00270: Cysteine and methionine metabolism" pathways were enumerated for each sample size between 5–600 as described in Fig. S1 legend. Dots and their horizontal arrays correspond to ratios (Y-axis) at specified sample sizes (X-axis).

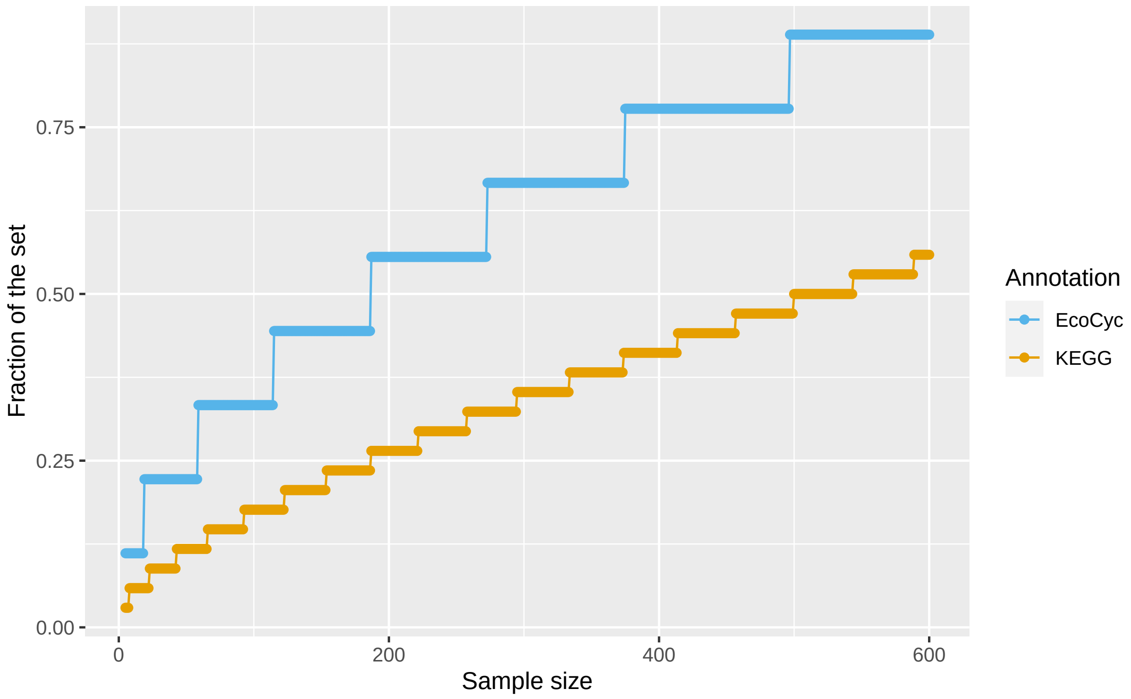

Figure S5: Fraction of the annotated pathway found in the critical subset. Critical subsets for “EcoCyc: superpathway of S-adenosyl-L-methionine biosynthesis” and “KEGG: eco00270: Cysteine and methionine metabolism” pathways were enumerated for each sample size between 5–600 as described in Fig. S1 legend. Dots and their horizontal arrays correspond to ratios (Y-axis) at specified sample sizes (X-axis). The fraction was calculated as a ratio of the critical subset size to the annotated set size.
