# Supplementary material for: Pathway size matters: the influence of pathway granularity on over-representation (enrichment analysis) statistics
Source: BMC Genomics. 2021 Mar 16;22:191. doi: 10.1186/s12864-021-07502-8 (PMC7967953; doi:10.1186/s12864-021-07502-8)
Supplement: Supplementary file 5 — Additional file 5 A listing of six KEGG maps and the multiple MetaCyc pathways those maps correspond to. [file 12864_2021_7502_MOESM5_ESM.pdf]

This file lists six KEGG maps and the multiple MetaCyc pathways that those maps correspond to.

1. KEGG map00230 Purine Metabolism

MetaCyc pathways:

5-aminoimidazole ribonucleotide biosynthesis I PWY-6121

inosine-5'-phosphate biosynthesis I PWY-6123

inosine-5'-phosphate biosynthesis II PWY-6124

inosine-5'-phosphate biosynthesis III PWY-7234

inosine 5'-phosphate degradation PWY-5695

sulfate activation for sulfonation PWY-5340

adenosine ribonucleotides de novo biosynthesis PWY-7219

guanosine ribonucleotides de novo biosynthesis PWY-7221

adenosine deoxyribonucleotides de novo biosynthesis I PWY-7227

adenosine deoxyribonucleotides de novo biosynthesis I PWY-7220

guanosine deoxyribonucleotides de novo biosynthesis I PWY-7226

guanosine deoxyribonucleotides de novo biosynthesis II PWY-7222

adenine salvage PWY-6610

adenine and adenosine salvage I P121-PWY

adenine and adenosine salvage II PWY-6605

adenine and adenosine salvage III PWY-6609

adenine and adenosine salvage V PWY-6611

adenine and adenosine salvage VI PWY-6619

guanine and guanosine salvage I PWY-6620

guanine and guanosine salvage II PWY-6599

guanine and guanosine salvage III PWY-6618

purine deoxyribonucleosides salvage PWY-7224

xanthine and xanthosine salvage SALVPURINE2-PWY

purine ribonucleosides degradation PWY0-1296

adenosine nucleotides degradation I	PWY-6596
guanosine nucleotides degradation II	PWY-6606
guanosine nucleotides degradation III	PWY-6608
purine deoxyribonucleosides degradation I	PWY-7179
purine deoxyribonucleosides degradation II	PWY-7179-1
ppGpp metabolism	PPGPPMET-PWY
glycolysis (only 2.7.1.40)	GLYCOLYSIS

2. KEGG map00130 Ubiquinone and other terpenoid-quinone biosynthesis

MetaCyc pathways:

phenylpropanoid biosynthesis, initial reactions PWY1F-467

4-hydroxybenzoate biosynthesis II (bacteria) PWY-5755

4-hydroxybenzoate biosynthesis III (plants) PWY-6435

3-(4-hydroxyphenyl)pyruvate biosynthesis PWY-5886

vitamin E biosynthesis (tocopherols) PWY-1422

vitamin E biosynthesis (tocotrienols) PWY-7436

plastoquinol-9 biosynthesis I PWY-1581

2-carboxy-1,4-naphthoquinol biosynthesis PWY-5837

phylloquinol biosynthesis PWY-5027

demethylmenaquinol-6 biosynthesis I/demethylmenaquinol-8 biosynthesis I/ demethylmenaquinol-9 biosynthesis PWY-5853/PWY-5852/ PWY-5851

menaquinol-6 biosynthesis/menaquinol-8 biosynthesis/ menaquinol-9 biosynthesis PWY-5849/MENAQUINONESYN-PWY/ PWY-5844

ubiquinol-6 biosynthesis (late decarboxylation)/ ubiquinol-7 biosynthesis (late decarboxylation)/ ubiquinol-8 biosynthesis (late decarboxylation)/ ubiquinol-9 biosynthesis (late decarboxylation)/ ubiquinol-10 biosynthesis (late decarboxylation) PWY3O-19/ PWY-5873/PWY-5870/PWY-5871/PWY-5872

ubiquinol-7 biosynthesis (early decarboxylation)/ ubiquinol-8 biosynthesis (early decarboxylation)/ ubiquinol-9 biosynthesis (early decarboxylation)/ ubiquinol-10 biosynthesis (early decarboxylation) PWY-5855/PWY-6708/PWY-5856/PWY-5857

shikonin biosynthesis PWY-5701

3. KEGG map00380 Tryptophan metabolism

MetaCyc pathways:

serotonin and melatonin biosynthesis PWY-6030

serotonin degradation PWY-6313

indole-3-acetate biosynthesis I PWYDQC-4

indole-3-acetate biosynthesis II PWY-581

L-tryptophan degradation II (via pyruvate) TRYPDEG-PWY

L-tryptophan degradation IV (via indole-3-lactate) TRPKYNCAT-PWY

L-tryptophan degradation VI (via tryptamine) PWY-3181

L-tryptophan degradation VIII (to tryptophol) PWY-5081

L-tryptophan degradation XI (mammalian, via kynurenine) PWY-6309

glucosinolate biosynthesis from tryptophan PWY-601

glutaryl-CoA degradation PWY-5177

L-tryptophan degradation to 2-amino-3-carboxymuconate semialdehyde PWY-5651

4. KEGG map00350 Tyrosine metabolism

MetaCyc pathways:

L-tyrosine degradation I TYRFUMCAT-PWY

L-tyrosine degradation II PWY-5151

L-dopa and L-dopachrome biosynthesis PWY-6481

eumelanin biosynthesis PWY-6498

pheomelanin biosynthesis PWY-7917

catecholamine biosynthesis PWY66-301

noradrenaline and adrenaline degradation PWY-6342

thyroid hormone biosynthesis PWY-6241

rosmarinic acid biosynthesis II PWY-5049

gentisate degradation I PWY-6223

gentisate degradation II PWY-7469

4-hydroxyphenylacetate degradation 3-HYDROXYPHENYLACETATE-DEGRADATION-PWY

5. KEGG map00330 Arginine and proline metabolism

MetaCyc pathways:

L-arginine degradation I (arginase pathway) ARGASEDEG-PWY

L-arginine degradation II (AST pathway) AST-PWY

L-arginine degradation III (arginine decarboxylase/agmatinase pathway) PWY0-823

L-arginine degradation IV (arginine decarboxylase/agmatine deiminase pathway) ARGDEG-III-PWY

L-arginine degradation VI (arginase 2 pathway) ARG-PRO-PWY

L-arginine degradation VII (arginase 3 pathway) ARG-GLU-PWY

L-arginine degradation VIII (arginine oxidase pathway) ARGDEG-IV-PWY

L-arginine degradation IX (arginine:pyruvate transaminase pathway) PWY-5742

L-arginine degradation X (arginine monooxygenase pathway) ARGDEG-V-PWY

L-proline biosynthesis I (from L-glutamate) PROSYN-PWY

creatine biosynthesis GLYCGREAT-PWY

creatine phosphate biosynthesis PWY-6158

creatinine degradation I CRNFORCAT-PWY

creatinine degradation II PWY-4722

nitric oxide biosynthesis I (plants) PWY-6845

nitric oxide biosynthesis III (bacteria) PWY-7860

putrescine degradation I PUTDEG-PWY

putrescine degradation II PWY0-1221

putrescine degradation III PWY-0

putrescine degradation IV PWY-2

putrescine degradation V PWY-3

spermidine biosynthesis II PWY-6559

spermine biosynthesis ARGSPECAT-PWY

spermine and spermidine degradation I PWY-6117

homocarnosine biosynthesis PWY66-421

6. KEGG map00240 Pyrimidine metabolism

MetaCyc pathways:

UMP biosynthesis I PWY-5686

UMP biosynthesis II PWY-7790

UMP biosynthesis III PWY-7791

UTP and CTP de novo biosynthesis PWY-7176

pyrimidine deoxyribonucleotides de novo biosynthesis I PWY-7184

UTP and CTP dephosphorylation I PWY-7185

UTP and CTP dephosphorylation II PWY-7177

pyrimidine deoxyribonucleotides de novo biosynthesis I PWY-7184

pyrimidine deoxyribonucleotides de novo biosynthesis II PWY-7187

pyrimidine deoxyribonucleotides de novo biosynthesis III PWY-6545

pyrimidine deoxyribonucleotides de novo biosynthesis IV PWY-7198

CMP phosphorylation PWY-7205

pyrimidine deoxyribonucleosides salvage PWY-7199

pyrimidine deoxyribonucleotides biosynthesis from CTP PWY-7210

pyrimidine nucleobases salvage I PWY-7183

pyrimidine nucleobases salvage II PWY-7194

pyrimidine ribonucleosides salvage I PWY-7193

pyrimidine ribonucleosides salvage II PWY-6556

pyrimidine ribonucleosides salvage III PWY-7195

pyrimidine deoxyribonucleosides degradation PWY-7181

pyrimidine deoxyribonucleotides dephosphorylation PWY-7206

uracil degradation I (reductive) PWY-3982

uracil degradation II (oxidative) PWY-6426

uracil degradation III PWY0-1471

thymine degradation PWY-6430

pyrimidine ribonucleosides degradation PWY0-1295
